# Socio-Demographic and Mental Health Profile of Admitted Cases of Self-Inflicted Harm in the US Population

**DOI:** 10.3390/ijerph15010077

**Published:** 2018-01-05

**Authors:** Chris Hanuscin, Golara Zahmatkesh, Anaheed Shirazi, Deyu Pan, Senait Teklehaimanot, Shahrzad Bazargan-Hejazi

**Affiliations:** 1 Department of Psychiatry, Charles R. Drew University of Medicine and Science, Los Angeles, CA 90059, USA; chanusci@gmail.com (C.H.); golarazahmatkesh@cdrewu.edu (G.Z.); anaheed.shirazi@gmail.com (A.S.); deyupan@cdrewu.edu (D.P.); 2 Department of Family Medicine, Charles R. Drew University of Medicine and Science, Los Angeles, CA 90059, USA; senaitteklehaimanot@cdrewu.edu; 3 David Geffen School of Medicine, University of California, Los Angeles, CA 90059, USA

**Keywords:** self-inflicted harm, race, psychiatric disorder, substance abuse, mental health, national trauma data bank

## Abstract

Self-inflicted harm (SIH) has a substantial lifetime prevalence, it is associated with tremendous costs, and its rate is increasing on a national scale. To examine the characteristics of those admitted for SIH in the US and to investigate the factors that potentially modify the methods used for SIH. This was a retrospective analysis of admitted cases of SIH including suicide attempts between 2007 and 2012 using the National Trauma Data Bank. We included a total of 204,633 cases admitted for SIH. Our participants were 75.1% males. Those aged 15–24 (21%), 25–34 (22%), 35–44 (19%), 45–54 (19%), and 55–64 (10%) years comprised the largest age groups among our cases—70.8%, 11.5%, 11.1%, and 6.6% were, respectively, Caucasians, Hispanics, Blacks, and Asian/Others. Analyses of the SIH methods revealed that Blacks were less likely to self-poison [Odds Ratio (OR): 0.78] compared to Whites, whereas individuals with psychiatric disorders or substance abuse carried 2.5 and 2.0-fold higher risk, respectively. Blacks were also less likely to use anoxic methods (OR: 0.69), whereas patients with psychiatric disorders or substance abuse carried 1.5-fold higher risk. Being Black, Hispanic, and Asian (OR: 0.58, 0.55, and 0.55, respectively) as well as having psychiatric disorders (OR: 0.80) were associated with lower risks of using firearms, whereas its risk was increased with increasing age. Blacks (OR: 0.77) were less likely to cut or pierce in contrast to Hispanics (OR: 1.4), Asians/Others (OR: 1.29), and those with psychiatric disorders (2.5-fold higher risk) or drug abuse (2-fold higher risk). Blacks (OR: 1.11), Hispanics (OR: 1.13), and Asians/Others (OR: 1.57) were more likely to jump from high places, whereas those with substance abuse were less likely (OR: 0.77). Among patients admitted for SIH, males, those aged 15–64 years, and Whites comprised the largest sex, age, and racial/ethnic groups, respectively. We also found that several factors including race/ethnicity, gender, age, and having concurrent psychiatric or drug abuse disorders can potentially influence the methods used for SIH.

## 1. Introduction

Self-inflicted harm (SIH), also known as self-inflicted injury, is an attempt to alter a disturbing mood state by inflicting serious harm to the body, e.g., by means of cutting, scratching, burning, or poisoning [1,2]. The lifetime prevalence of SIH is reportedly 2.5–6% [2,3]. Studies of national trends report increasing hospital admissions due to SIH as well as a higher likelihood of concomitant mental disorders in SIH cases [4,5]. SIH emergency department (ED) visits were over three million between 2006 and 2013 [6]. In 2010 alone, there were 316,572 hospitalizations due to SIH with over $3.5 billion in medical costs and $6.3 billion in productivity loss, according to the Centers for Disease Control and Prevention (CDC) data [7]. However, the real costs associated with SIH are higher considering the large burden of suicide thoughts and attempts in individuals who do not seek treatment at all. Notably, a quarter of suicides are preceded by nonfatal SIH in the prior year [8]. Suicide is currently the 10th leading cause of death in the United States (US), its incidence is substantially increased from 26,869 in 1980 to 42,773 in 2014, and it is projected to become a leading cause of death by 2030 [7,9]. The above staggering numbers highlight the importance of SIH and the tremendous need to better characterize it in order to devote the required resources to the populations at risk.

The Nationwide Emergency Department Sample data showed that SIH was more common in females (57.4%), its incidence peaked at the age of 15 to 19 years, and its average age of presentation was 33 years [6]. The most common mechanisms of injury were poisoning (66–68%) followed by cutting and penetrating injuries (20–22%). Of note, over 80% of SIH cases had a concurrent mental disorder [6,10]. Our prior study of SIH cases in Los Angeles County revealed that African Americans had the highest SIH admission rates when compared to the populations at risk, followed by Whites, Latinos, and Asians [11]. Medicare/Medicaid was the most common type of health coverage among African Americans, whereas Whites and Asians most commonly had private insurance coverage [11]. That study was among the first investigations of the racial/ethnic disparities in SIH admissions. However, this topic requires further investigations at the national level.

In this study, we aimed to: (1) examine the socio-demographic and mental health profile of hospitalized SIH cases in the U.S. population; and (2) test the links between race/ethnicity and SIH methods in this population controlling for insurance coverage and mental disorders. 

## 2. Methods

### 2.1. Data Source and Participants

This was a retrospective analysis of the National Trauma Data Bank (NTDB)—sponsored by the American College of Surgeons Committee on Trauma and the Center for Disease Control and Prevention (CDC)—between 2007 and 2012. The databank was originally created to develop a hospital trauma registry as a standard reference database of seriously injured patients for research and monitoring purposes. Of note, data submission to this database is voluntary; however, it is the largest aggregation of U.S. trauma registry data ever assembled. Registry data that is collected from the NTDB is compiled annually and disseminated in the forms of hospital benchmark reports, data quality reports, and research data sets. Access to data sets is available to researchers who apply and are approved [12]. We included the hospitalized trauma cases due to SIH including suicide attempts with or without a concurrent psychiatric diagnosis i.e., schizophrenic disorders, episodic mood disorders, and non-organic psychosis. Notably, those who passed away due to SIH or suicide were not excluded.

### 2.2. Study Variables

We used the following the International Classification of Diseases, Ninth Revision, ICD-9 codes to identify SIH: E950–952 for suicide or self-inflicted poisoning by solid or liquid substances, gases in domestic use, or other gases and vapors; E953–954 for suicide or SIH by hanging, strangulation, suffocation, or submersion (drowning); E955 for suicide or SIH by firearms, air guns or explosives; E956 for suicide or SIH by cutting and piercing instruments; E957 for suicide or SIH by jumping from high places; and E958 for suicide or SIH by other or unspecified means. We also used ICD-9 codes 295, 296, and 298 to identify concurrent psychiatric disorders, i.e., schizophrenic disorders, episodic mood disorders, and non-organic psychosis; and ICD-9 code 303–305 to identify substance abuse [11].

We extracted the data on race/ethnicity, age ranges, gender, and insurance status. Specifically, we used the documented races White, Black, Hispanic, and Asian/Other; and genders Male and Female. Lastly, we consolidated private insurance categories such as Blue Cross/Blue Shield, Automobile, private or commercial insurances, and worker’s compensation into a single category of Private Insurance. Medicare, Medicaid, and other government insurance categories were classified into the Government Insurance category. To clarify, there is a multi-payer health coverage system in the U.S. in which private insurances are usually available through employers in contract with the private insurance companies; Medicare is available through the government for those ≥65 years old or with end-stage renal disease; Medicaid is also available through the government but for underprivileged individuals; and self-pay insurance is available through state marketplaces to be purchased during the open enrollment periods.

### 2.3. Data Analysis

In addition to descriptive analyses, we used unadjusted and adjusted multinomial logistic regression analysis to investigate the associations between the SIH methods and race/ethnicity, controlling for mental disorders and other demographic variables. We compared the likelihood of engaging in each SIH method in various racial/ethnic groups to their White counterparts. Other/unspecified means of SIH were used as a reference group for SIH methods. In all analyses, *p* < 0.05 or 95% confidence intervals with no overlap with the null effect value was considered statistically significant. We used IBM SPSS Statistics v22.0 (IBM Corp., Armonk, NY, USA) for data analyses.

## 3. Results

### 3.1. Characteristics of the Study Participants

**Figure A1:**
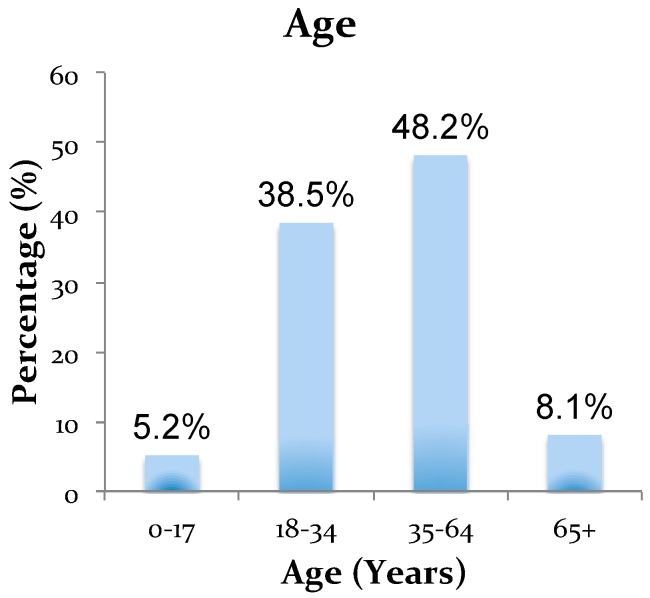
Age differences among self-inflected harm (SIH) cases.

**Figure A2:**
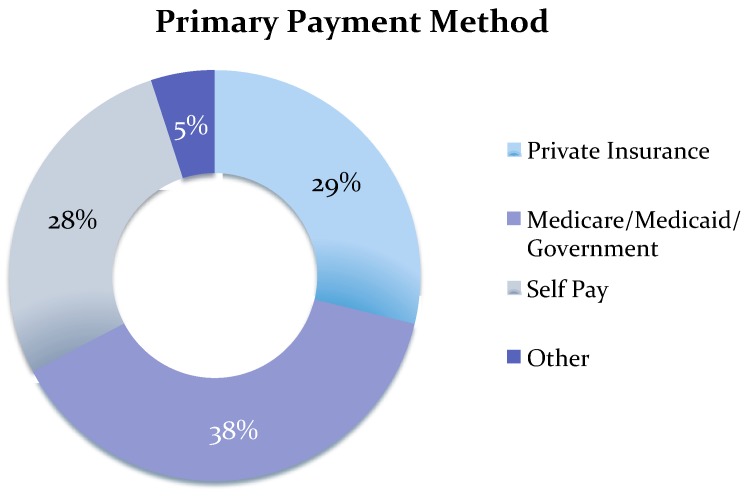
Primary Payment Method differences among SIH cases.

**Figure A3:**
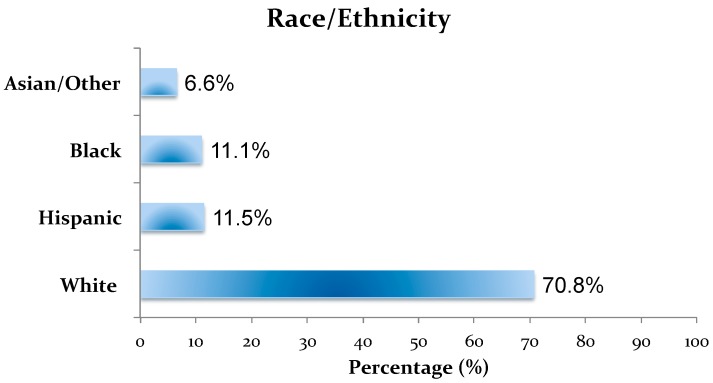
Race and Ethnicity differences among SIH cases.

We analyzed a total of 204,633 SIH cases. The proportion of missing data was considerable for a number of the studied variables. Notably, 45%, 44%, 33%, and 43% of the data were missing on race/ethnicity, gender, age, and insurance types. Socio-demographic characteristics of the study participants are outlined in Table 1 and Appendix A Figure A1, Figure A2 and Figure A3.

**Figure A4:**
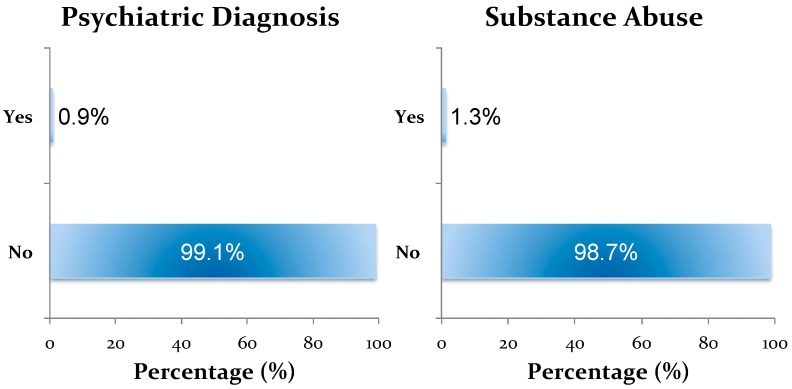
(**Left**) % of participants with and without psychiatric disorders; (**Right**) % of participants with and without psychiatric disorders.

Among our participants, 202,782 (99.1%) did not have any concurrent psychiatric disorder, whereas 1851 (0.9%) did. In addition, 201,894 (98.7%) did not have a concurrent substance abuse disorder, while 2739 (1.3%) did (Figure A4).

Out of all 204,633 cases, the usage of firearms was the most common method for SIH (74,577; 36.44%). This was followed by cutting and piercing techniques (58,344; 28.51%), jumping from high places (37,008; 18.09%), other/unspecified methods (25,751; 12.58%), anoxic injuries i.e., those caused of deprivation of adequate oxygen (7081; 3.46%), and poisoning (1872; 0.91%). This trend and preference order remained constant throughout the six-year period of our study (Table 2).

### 3.2. Results of Multinomial Logistic Regression Analyses

**Figure 1:**
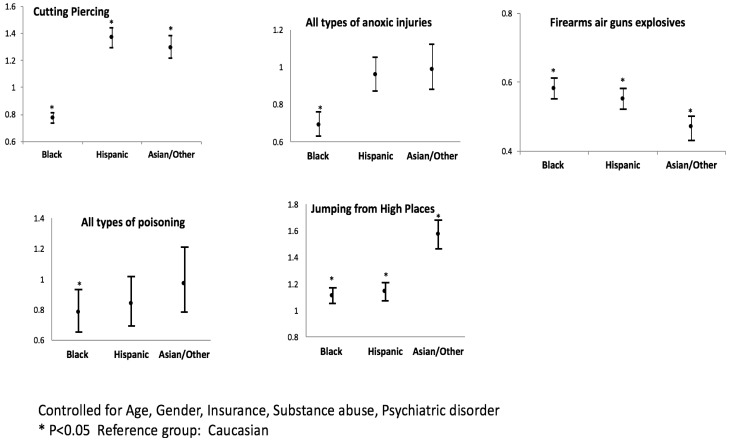
Association of race/ethnicity with various methods of self-inflicted injury (graphs show odds ratio with their 95% confidence intervals).

We observed that, compared to Whites, Blacks were less likely to use cutting/piercing as a form of SIH, whereas Hispanics and Asians/others were more likely to use this method. Also compared to Whites, being Black was associated with a lower risk of using anoxic methods of SIH. Additionally, Blacks, Hispanics, and Asians/others were less likely to engage in using firearms for SIH. We also observed that, compared to Whites, Blacks were less likely to use poisoning as a form of SIH. Conversely, being Black, Hispanic, or Asian/other was found to be a risk factor for jumping from high places (Table 3 and Figure 1).

Females were more likely to engage in self-poisoning, and they were less likely to use cutting/piercing, anoxic methods, or firearms compared to males. Older age groups had higher odds of using firearms but lower odds of using anoxic methods or jumping from high places; for other methods, age did not appear to be an influencing factor. Having government coverage (Medicare/Medicaid) or self-pay insurance was associated with higher odds of engaging in cutting or piercing; in addition, having government coverage was associated with higher odds of engaging in anoxic methods and jumping from high places, whereas having self-pay coverage was associated with higher odds of using firearms (Table 3).

In our study, those with psychiatric disorders or substance abuse were more likely to engage in cutting/piercing, using anoxic methods, or self-poisoning, whereas they were less likely to use firearms for SIH. Lastly, patients with substance abuse were less likely to jump from high places (Table 3).

## 4. Discussion

In summary, we found that, among those admitted for SIH in the US, (A) males were more frequent than females; (B) those aged 15–64 years comprised the most cases; (C) Whites comprised the largest number of SIH admissions in our study, followed by Hispanics, Blacks, and Asians; (D) a small proportion concurrently had psychiatric or drug use disorders; (E) there were significant racial/ethnic disparities in the methods used for SIH; and (F) other factors such as sex, age, type of insurance, and having concurrent psychiatric or drug abuse disorders probably modify the methods used for SIH.

In our study, males comprised a larger proportion of SIH admissions in contrast to other studies: in a study of nationwide trends in the suicide-related ED visits in the US from 2006 to 2013, females comprised a larger proportion (57.4%) than males (42.6%) [6]. Similarly, females comprised the majority of cases in another study of SIH admissions in Los Angeles County by Bazargan et al. [11] as well as in four other studies [4,13,14,15]. This can be due to the fact that we studied the admitted patients who probably had more serious injuries. The biggest proportion of our SIH cases were aged 15–64 years. Olfson et al. [5] showed that the rate of hospital-treated self-harm events increased from 5.1 (2001) to 7.1 (2011) per 10,000 individuals among middle-aged adults (aged 45–64 years), which was the largest increase among all age groups. 

Whites comprised the largest number of SIH admissions in our study, followed by Hispanics, Blacks, and Asians. In a study by Vaughn et al. [16], Caucasians were more likely to report SIH. Nevertheless, the larger number of Whites in our study most likely represents the racial/ethnic composition of the US population: in our study, Whites, Blacks, Hispanics and Asians/Others respectively comprised 70.8%, 11.1%, 11.5%, and 6.6% of the admitted SIH cases; this composition was similar to the racial/ethnic composition of the US population consisting of 76.9% Whites, 13.3% Blacks, 17.8% Hispanics, and 5.9% Asians/Others (total >100% as some people identify as >1 race/ethnicity) [17].

We found significant racial/ethnic disparities in the methods used for SIH: Blacks were less likely to use cutting/piercing, anoxic methods, poisoning or firearms. Whites, however, were most likely to engage in using firearms. Previous reports suggest that Caucasians are more likely to inflict self-harm using gunshots, whereas Blacks are more likely to be victims of cranial gunshot assaults [18]. Similarly, Whites had slightly higher rates of non-fatal self-harm poisoning than Blacks and significantly higher rates than Hispanics between 2001 and 2004 in a study by Prosser et al. [19]. In this study, females were more likely to do self-poisoning. Additionally, we observed that being Black, Hispanic or Asian was associated with an increased risk of jumping from high places. This finding is not reported by any prior study to the best of our knowledge. Prior studies reported self-poisoning to be the most common method of SIH across all racial/ethnic subgroups [11,20,21,22,23]. In a U.K. study, self-poisoning comprised above 80% of SIH incidents [24]. However, firearm usage was the most common SIH method in our study followed by cutting/piercing, jumping from high places, other/unspecified methods, anoxic injuries, and poisoning. Again, this discrepancy is most likely because we studied the admitted patients who probably had more serious injuries, and unintentionally excluded many cases of self-poisoning and minor self-cutting episodes.

Among our participants, only 1.3% were diagnosed with substance abuse and less than 1% with psychiatric comorbidities. In contrast, Bazargan et al. [11] observed that over 40% of their participants were dealing with substance abuse, over 8% with alcoholism, and considerable proportions with various mental disorders. In their study, episodic mood disorders (49.3%) were the leading concurrent psychiatric disorders followed by depressive disorders (19.2%), anxiety disorders (11.2%), and schizophrenia (10.8%). Mental health issues and alcohol use were also common in a multicenter study of 24,598 cases of self-harm in England from 2000–2010, particularly in those aged 35–54 years and those with repeated self-harm [25]. We did not detect concurrent psychiatric disorders in a substantial majority of our participants; however, we observed a significant association between having psychiatric disorders or substance abuse with doing certain methods of SIH such as cutting themselves, consuming poisons, and using anoxic methods. The scarcity of the concurrent psychiatric disorders in our sample is most likely due to an underdetection as many clinicians who collected data were likely focusing on the medical problems leading to missing the psychiatric diagnoses in many included patients.

As a retrospective analysis of the NTDB data, our study possesses the inherent limitations of a retrospective observational study. We were not directly involved in the primary data collection for this study, and, therefore, we cannot rule out errors in data collection or coding. In addition, the data is collected via convenient sampling rather than probabilistic population-based sampling methods. In addition, the large volume of missing data for some variables limits the internal and external validities of our study. Moreover, the data is voluntarily submitted by a disproportionate number of larger hospitals committed to monitoring and improving the care of younger and severely injured patients. Furthermore, we analyzed hospitalized cases rather than those presented to the ED; thus, our results generally represented those with more severe injuries requiring hospitalization, and they cannot be extrapolated to all SIH cases. Nevertheless, we analyzed a large sample of SIH cases to provide new key insights regarding patients admitted for SIH.

## 5. Conclusions

We observed that, among patients admitted for SIH, males, those aged 15–64 years, and Whites comprised the largest sex, age, and racial/ethnic groups. We also found that several factors including race/ethnicity, gender, age, type of insurance, and having concurrent psychiatric or drug abuse disorders can potentially influence the methods used for SIH. Further studies are needed to verify our results, expand on the characteristics of SIH beyond the admitted cases in the US and other countries, and design and test the interventions targeting the most vulnerable populations and reducing the SIH rates at local, regional, and national scales.

## Figures and Tables

**Table 1:** Socio demographic characteristics of study participants (2007–2012).

		Total in SIH Cases* N*	SIH Cases %
**Race/Ethnicity**	White	122,606	71.6
	Black	19,076	11.1
	Hispanic	18,525	10.8
	Asian/Other	11,050	6.5
**Gender**	Male	154,373	75.8
	Female	49,283	24.2
**Age**	0–14 years	2902	1.4
	15–24 years	42,826	21.0
	25–34 years	45,059	22.1
	35–44 years	38,367	18.9
	45–54 years	37,783	18.6
	55–64 years	20,709	10.2
	65–74 years	8844	4.4
	75–84 years	5415	2.7
	85+ years	1508	0.7
**Insurance**	Private Insurance	52,583	25.7
	Government Insurance	67,519	33.0
	Self-pay	49,141	24.0
	Other	35,390	17.3

SIH: self-inflected harm.

**Table 2:** Trend of methods to do self-inflicted harm from 2007 to 2012.

Year	Poisoning	Anoxic	Firearms	Cut/Pierce	Jumping	Other
2007	249 (1.06)	904 (3.83)	8848 (37.49)	6346 (26.89)	4372 (18.53)	2880 (12.20)
2008	299 (1.00)	831 (2.78)	10,971 (36.71)	8933 (29.89)	5214 (17.45)	3640 (12.18)
2009	321 (0.99)	1118 (3.43)	12,242 (37.57)	9236 (28.34)	5629 (17.27)	4039 (12.40)
2010	312 (0.88)	1200 (3.39)	12,798 (36.19)	10,038 (28.39)	6484 (18.34)	4530 (12.81)
2011	260 (0.65)	1407 (3.53)	14,576 (36.61)	11,435 (28.72)	7256 (18.22)	4881 (12.26)
2012	431 (0.99)	1621 (3.74)	15,142 (34.90)	12,356 (2848)	8053 (18.56)	5781 (13.33)
Total	1872 (0.91)	7081 (3.46)	74,577 (36.44)	58,344 (28.51)	37,008 (18.09)	25,751 (12.58)

**Table 3:** Results of multinomial logistic regression analysis.

Variables	Odds Ratios	Confidence Intervals
**Cutting/Piercing**		
15–24 years	1.36	1.23–1.51
25–34 years	1.66	1.50–1.83
35–44 years	1.81	1.64–1.99
45–54 years	1.73	1.56–1.91
55–64 years	1.63	1.47–1.81
65–74 years	1.21	1.09–1.36
75–84 years	0.99	0.87–1.11
85+ years	1.29	1.10–1.51
Female	1.13	1.11–1.16
Black	0.98	0.95–1.02
Hispanic	1.72	1.67–1.78
Asian/Other	1.51	1.45–1.57
Government Insurance	1.25	1.21–1.29
Self-Pay Insurance	1.12	1.09–1.16
Other Insurance	1.14	1.10–1.18
Psychiatric Disorder	2.46	2.42–2.96
Substance Abuse	2.22	2.05–2.43
**Anoxic Injuries**		
15–24 years	0.28	0.25–0.32
25–34 years	0.24	0.21–0.27
35–44 years	0.26	0.23–0.29
45–54 years	0.19	0.17–0.22
55–64 years	0.12	0.10–0.14
65–74 years	0.06	0.05–0.08
75–84 years	0.06	0.04–0.08
85+ years	0.08	0.05–0.12
Female	0.78	0.74–0.84
Black	0.87	0.80–0.94
Hispanic	1.01	0.93–1.10
Asian/Other	0.98	0.88–1.09
Government Insurance	1.15	1.08–1.23
Self-Pay Insurance	0.97	0.90–1.05
Other Insurance	1.09	1.00–1.19
Psychiatric Disorder	1.1	0.84–1.43
Substance Abuse	1.16	0.94–1.44
**Firearms**		
15–24 years	0.92	0.84–1.00
25–34 years	0.87	0.80–0.96
35–44 years	0.9	0.82–0.99
45–54 years	1.16	1.06–1.26
55–64 years	1.55	1.41–1.70
65–74 years	3.45	3.12–3.81
75–84 years	4.64	4.17–5.17
85+ years	3.3	2.86–3.81
Female	0.5	0.49–0.51
Black	0.66	0.64–0.68
Hispanic	0.47	0.46–0.49
Asian/Other	0.37	0.35–0.39
Government Insurance	0.7	0.68–0.72
Self-Pay Insurance	1.18	1.14–1.21
Other Insurance	0.85	0.82–0.88
Psychiatric Disorder	0.47	0.42–0.54
Substance Abuse	0.68	0.62–0.75
**Poisoning**		
15–24 years	0.43	0.33–0.55
25–34 years	0.25	0.19–0.33
35–44 years	0.4	0.31–0.52
45–54 years	0.4	0.31–0.53
55–64 years	0.46	0.35–0.61
65–74 years	0.25	0.17–0.36
75–84 years	0.26	0.17–0.41
85+ years	0.53	0.30–0.93
Female	2.57	2.33–2.84
Black	0.98	0.83–1.15
Hispanic	0.83	0.69–1.00
Asian/Other	0.96	0.78–1.17
Government Insurance	1.02	0.90–1.15
Self-Pay Insurance	0.85	0.74–0.99
Other Insurance	0.79	0.66–0.94
Psychiatric Disorder	1.89	1.30–2.75
Substance Abuse	1.46	1.00–2.12
**Jumping from High Places**		
15–24 years	1.5	1.35–1.67
25–34 years	1.52	1.37–1.69
35–44 years	1.32	1.18–1.47
45–54 years	1.17	10.5–1.31
55–64 years	0.88	0.79–0.99
65–74 years	0.48	0.42–0.55
75–84 years	0.42	0.36–0.49
85+ years	0.31	0.24–0.40
Female	1.53	1.49–1.57
Black	1.54	1.49–1.60
Hispanic	1.23	1.18–1.28
Asian/Other	1.83	1.74–1.91
Government Insurance	1.28	1.24–1.32
Self-Pay Insurance	0.76	0.73–0.79
Other Insurance	1.06	1.02–1.10
Psychiatric Disorder	0.62	0.53–0.72
Substance Abuse	0.52	0.46–0.60
